# A care coordinator screening strategy to address health harming legal needs

**DOI:** 10.1186/s12913-021-07440-x

**Published:** 2022-02-16

**Authors:** Daniel Berg, Alice Setrini, Kathy Chan, Ann Cibulskis, Kulsum Ameji, Keiki Hinami

**Affiliations:** 1 Cook County Health, 1950 Polk St, Suite 5800, Chicago, 60612 IL USA; 2 Legal Aid Chicago, 120 S LaSalle St Ste 900, Chicago, 60603 IL USA; 3 Chicago Department of Public Health, 333 S State St. #200, Chicago, 60604 IL USA

**Keywords:** Care coordination, Health Harming Legal needs, Medical Legal Partnership, Patient navigators

## Abstract

**Background:**

Medical legal partnerships provide an opportunity to help address various social determinants of health; however, the traditional practice of screening patients during clinical encounters is limited by the capacity of busy clinicians. Our medical legal partnership utilized care coordinators trained by the legal service attorneys to screen patients outside of clinical encounters for health harming legal needs. The goal of our study was to demonstrate that our novel model could successfully identify and refer patients of a safety-net healthcare system to appropriate legal services.

**Methods:**

We conducted a mixed methods evaluation of the program. Data was collected during the implementation period of the program from March 2017 to August 2018. Operational data collected included number of patients screened, number of referrals to the legal partner, source and reason for referrals. Return on investment was calculated by subtracting program costs from the total reimbursement to the health system from clients’ insurance benefits secured through legal services.

**Results:**

During the 18-month study, 29,268 patients were screened by care coordinators for health harming legal needs, with 492 patients (1.7%) referred for legal assistance. Of the 133 cases closed in 2017, all clients were invited to participate in a telephone interview; 63 pre-consented to contact, 33 were successfully contacted and 23 completed the interview. The majority (57%) reported a satisfactory resolution of their legal barrier to health. This was accompanied by an improvement in self-reported health with a decrease of patients reporting less than optimal health from 16 (89%) prior to intervention to 8 (44%) after intervention [risk ratio (95% confidence interval): 0.20 (0.04, 0.91)]. Patients also reported improvements in general well-being for themselves and their family. The healthcare system recorded a 263% return on investment.

**Conclusions:**

In our medical legal partnership, screening for health harming legal needs by care coordinators outside of a clinical encounter allowed for efficient screening in a high risk population. The legal services intervention was associated with improvements in self-reported health and family well-being when compared to previous models. The return on investment was substantial.

## Background

By one estimate, the number of deaths attributable to the social determinants of health including low education, racial segregation, low social support, individual-level poverty, and income inequality is comparable to the number of deaths attributable to the leading bio-medical causes of death in the United states including myocardial infarction, cerebrovascular disease, and lung cancer [1]. According to the landmark 2017 Justice Gap Report, low income households are especially impacted by legal barriers, with 71% of low income households having at least one civil legal problem and 41% of low-income households estimated to have civil legal needs associated with health care [2]. Fewer than one in five legal problems experienced by low income individuals are addressed with the help of an attorney who understands how to successfully navigate the legal system [3], and many vulnerable patients cannot ascertain their legal needs [4]. Some of these legal needs such as access to appropriately maintained housing, adequate food, and even applying for state funded insurance such as Medicaid can improve health. To address these health harming legal needs, Medical Legal Partnerships (MLPs), pioneered in the 1990 s, recruit the capacities of clinicians and lawyers to directly address legal-barriers to health [5] and more than 300 hospitals around the United States have adopted an MLP program [6].

Most described MLPs in the United States are based in a health care clinic (either primary care or specialty care) and patients are screened by either a physician or a member of the staff in the clinic during a routine clinic visit. Positive screens trigger detailed interviews through which the patient’s specific health harming legal situation is understood as that requiring the intervention of a legal professional. Limitations of time, training, and human resources may cause health care organizations to inadequately identify and address patients’ health harming legal needs. As many as 79% of health care organizations in the United States with an MLP reported screening for health harming legal needs among the social determinants of health. However, because the time and resources required to screen, assess, and refer legal needs are substantial, only 30% screened the general population “all of the time,” and only 63% of health care partners used a formal screening protocol [7].

Some programs utilize medical assistants or social workers to screen patients for health harming legal needs, but medical assistants in these models also juggle competing responsibilities [8] and many Licensed Clinical Social Workers spend an increasing amount of time on mental health counseling and less time on tasks associated with helping patients obtain social services and resources to assist patients with their medical needs [9]. Other programs have tried to integrate screening for health harming legal needs in the electronic medical record [10], or incorporate self-screening as part of the pre-visit check-in [3]. Care coordinators or navigators were used to conduct screening for health harming legal needs in the Whitman-Walker Health system [6] but only in a focused group of LGBTQ and HIV-positive communities utilizing a federally qualified health center.

Screening for health harming legal needs may be effectively conducted by care coordinators, whose cost-effectiveness in various areas is supported by recent studies [11, 12]. As care coordinators are not a part of the clinic staff they do not have the same time constraints as clinical staff or clinicians. This allowed us to add the patient service of screening and referral to the MLP without adding new responsibilities to primary and specialty care clinics that were already strained for time and resources. The use of care coordinators to screen, assess, and refer a general population of a safety-net population for legal services has not been described previously in literature. The goal of the study was to demonstrate that referrals from care coordinators screening patients outside of the clinic are comparable to referrals from clinical staff. We also evaluated outcomes using legal case status, patient self-reported health, general well-being of patient and family, and return on investment compared to previous studies.

### Medical legal Partnership at cook county health

Cook County Health (CCH) is a public healthcare system serving about 300,000 patients each year in 2 hospitals, 15 community health centers, and correctional health services in Cook County Jail and the Cook County Juvenile Temporary Detention Center. In any given year, approximately a third of CCH patients are uninsured and another third are Medicaid insured. CCH also administers CountyCare, the largest Medicaid managed care plan for Cook County Medicaid beneficiaries, with more than 330,000 members in 2018. To further the organization’s service mission and identify potential policy changes to decrease health disparities, an MLP was established and implemented in March 2017 through a partnership of Cook County Health, Legal Aid Chicago (formerly Legal Assistance Foundation (LAF)), and the Chicago Department of Public Health. Legal Aid Chicago is a civil legal services organization, providing bi-lingual (English/Spanish) non-criminal legal services to people living in poverty and other vulnerable populations. Initial funding for the partnership was provided through the BUILD Health Challenge Grant, which comes from a collaboration of private foundations seeking to improve coordination between community-based organizations, health systems, and local public health departments.

The MLP targeted various high-risk populations assigned to CCH’s complex care program for patients with profile similar to the highest utilizers of public entitlement programs [13]. Patients in the complex care program were assigned to a care coordinator. In addition to patients of the complex care program, care coordinators screened for the healthcare needs of approximately 4000 patients from May 2017 to August 2018 in Chicago’s Central Bond Court located on the premises of Cook County Jail prior to detainees’ court hearing. Finally, patients admitted to the hospital and seen in the emergency department represented some of the most medically vulnerable population screened by care coordinators.

Care coordinators are nurses, social workers, and community health workers who work directly with patients and are familiar with patient’s medical conditions, psychosocial circumstances, living environment, history, values and readiness for self-care management [14] in order to identify barriers to health and help patients overcome those barriers. Care coordinators interact with patients inside and outside health care facilities to identify how their disease impacts their social and emotional health, and how their distinct situations impact the overall health of the patient. They help patients coordinate care between primary care, specialty care and ancillary services (wound care, physical therapy, etc.) with the goal of reducing complications from chronic medical conditions. At CCH care coordinators screen patients for health and social needs using a standardized health risk screening tool which includes self-reported health care needs, social determinants including food, housing, and personal safety and assist patients with coordinating appointments and transportation.

Legal Aid Chicago worked closely with the care coordination team and provided continual and regular training to educate the care coordinators about what health harming legal needs are, how to identify them, which legal issues can be addressed through a high intensity legal intervention, and which issues are appropriate for self-advocacy. Between March 2017 and August 2018, 22 training sessions were held. Training used a mnemonic that identifies common social determinants of health known as I-HELP [15]. IHELP stands for Income, Housing, Education & Employment, Legal Status, Personal and Family Stability. All categories of IHELP were covered during the training sessions, but public benefits were identified as the highest need that could be addressed by this partnership.

Care coordinators were not given a script or specific questions to use for screening for health harming legal needs, but were given training about how to identify health harming legal needs during their regular patient interactions. Care coordinators were equipped to manage common social needs such as provision of information and referrals to social services. When care coordinators elicited health harming legal needs beyond their scope, they discussed the availability of legal resource with the patient, obtained permission to make a referral, and referred patients to Legal Aid Chicago through a warm handoff. Care coordinators were encouraged to consult with the project attorney if there was a question as to whether a patient’s issue was appropriate for a referral in order to ensure the quality of referrals through regular feedback. Commonly surfaced legal issues that were not appropriate for extended representation provided insight for where self-advocacy materials would be valuable for care coordinators to share directly with patients.

To effect the warm handoff, care coordinators communicated directly with the staff attorney providing a brief description of patients’ needs and their contact information. Legal Aid Chicago staff made at least 3 attempts to contact the patient; if those efforts were unsuccessful a letter was sent to the patient with information on how they can connect with the attorney directly if the legal issue persisted. After successful contact with the patient, the staff attorney performed an intake interview to determine the nature of the legal issue, ensure that no legal conflicts of interest existed, and develop their recommended course of action. Recommendations provided by the staff attorney after reviewing the case include: direct legal services provided by the staff attorney, referral to an internal legal specialty practice group, advice on self-advocacy measures, or referral to an outside agency. Once the case was closed, and permission from the client obtained, information about the outcome of the case was relayed back to the care coordinator who made the initial referral. This closed-loop provided continual reinforcement and refinement of the care coordinators knowledge of how to identify and refer health harming legal needs.

## Methods

We performed program evaluation using a mixed methods approach conducted in 2 parts. In the first part, operationally collected data about cases of health harming legal needs screened, referred, and managed were compiled into figures illustrating trends. Expenses from grants and investment from health care system were collected. We reviewed hospital reimbursement from insurance that was acquired through the MLP to determine return on investment.

The second part involved the assessment of patient-reported outcomes data during the program’s pilot phase in 2017. All 133 clients whose legal cases were closed in 2017 were informed about their eligibility to participate in a research study and those willing to participate provided pre-consent to be contacted. The 63 pre-consenting participants for the evaluation research were called by telephone to undergo a formal pre-scripted verbal informed consent procedure. Up to 3 telephone contact attempts over 1 month were made for each pre-consenting client, and 33 participants were contacted. We communicated verbally using standardized questionnaires, in English or Spanish per patient-preference, for the 23 patients who consented to evaluation.

We administered a custom questionnaire including the following constructs to consenting participants. General self-rated health, the Patient Health Questionnaire-2 (PHQ-2) [16], food insecurity (2-items) [17], hospital utilization over 12 months, and emergency department utilization over 6 months. Each of these constructs was chosen because it had been previously administered to each patient as part of the unmet legal needs screening and potential changes could be assessed using a pre-post study design. We used McNemar’s Chi2 test for assessing independence of paired data. In addition, we assessed the degree to which legal services had an impact “on your health”, “on your general well-being”, “on your family’s health”, and “on your family’s general well-being” using a response scale ranging from (-3) extremely negative, (-2) moderately negative, (-1) somewhat negative, (0) none, (+1) somewhat positive, (+2) moderately positive, to (+3) extremely positive. Responses to these questions were displayed as coordinate heat maps. In order not to presume a favorable patient experience from our intervention, we asked open-ended questions that allowed patients to report negative experiences. We asked how the legal intervention translated into a potential change in health status and solicited comments for how we could have done better. We performed thematic content analysis and selected representative comments.

## Results

**Fig. 1:**
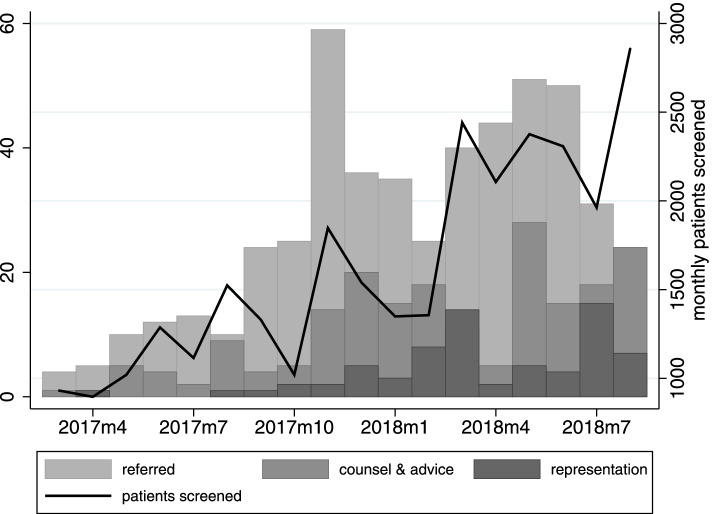
Patient screened and referred by CCHHS and Countycare Care Coordinators

During the 18-month study period from March 1 2017 to August 31 2018, 29,268 patients underwent screening for legal needs. Of the 492 patients referred to legal assistance, 97 (30%) received extended services involving legal representation. Figure 1 illustrates the increase in the monthly number of patients screened during the program’s implementation period, the number of patients referred for legal assistance, and those who required counsel and advice and those who required representation. In general, the number of patients referred to Legal Aid Chicago tracked around 1.7% of patients screened. The number of cases that required representation tended to increase through the study which may demonstrate improvements in care coordinators’ ability to identify complex cases suitable for referral.

Table 1 breaks out the legal problems and source of referrals. In total 191 patients were referred to Legal Aid Chicago in the 10 months from March to December 2017, and another 301 patients in the 8 months from January 1, 2018 to August 31, 2018. The majority of referrals were from the complex care program and second highest referral source was from patients admitted to the health system’s principal hospital. The highest category of need in the population of screening patient was for public benefits, with 57% of patient referrals in that category. Table 2 shows the resolution of the referrals. Since the first referrals in March 2017 until the end of August 2018, a total of 323 cases were closed. Of the closed cases, 226 cases did not require full representation, which include cases that were closed as advice or referred to another agency while 97 cases (30%) required full representation.

**Table 1:** Source and Legal Problems of health harming legal needs referred to MLP

	Mar 2017-Dec 2017	Jan 2018- Aug 2018
Patients Referred	191	301
Legal Problem Category		
Public Benefits	112	170
ADAPT	23	54
Family Law	15	25
Housing	13	23
Immigration	4	4
Consumer	6	6
Employment	4	1
Education	0	0
Other	14	18
Source of Referral		
Complex Care	108	202
Inpatient	52	45
Bond Court	19	8
Unassigned	12	46

*ADAPT* services are Advanced Directives, Powers of Attorney for Health and Transfer of Instruments

**Table 2:** Resolution of MLP Referrals

Resolution		
Closed	133	190
Rejected^a^	52	97
Open	6	14
Pending	0	0
Prescreen	0	0
Close Reason		
Counsel and Advice	87	121
Limited Action	17	20
Extensive Service	7	22
Referral without Advice^b^	6	12
Negotiated Settlement (with litigation)	3	7
Administrative Agency Decision	12	5
Negotiated Settlement (without litigation)	1	2
Contested Court Decision	0	1

^a^Rejected: No Show/No Contact, Caller no longer wants aid, outside priorities, Duplicate Case, Out of Legal Aid Chicago Service, Over income, Conflict of Interest, Other

^b^Over income/assets, undocumented, outside priorities

As shown in Table 3, our study did not detect changes in hospitalization or emergency department use for patients that participated in the interviews. However, we calculated a gross return on investment from reimbursements through insurance associated with patients involved in the medical legal partnership. The program planning cost was $75,000, operating cost was $100,000 for each calendar year of the study period. In comparison, the MLP helped the healthcare system recoup approximately $1 million in service charges from the state Medicaid program. The return on investment for the health system was thus calculated to be 263%. From a societal perspective, the nearly 300 patients who were linked to public benefits improved their financial position.

### Patient reported measures

Sixty-three patients pre-consented to be contacted for interview. Of 33 pre-consenting patients who were successfully contacted, 23 (70%) consented to the evaluation. The legal intervention was received favorably by the majority who interacted with Legal Aid Chicago services with 13 (57%) agreeing or somewhat agreeing with the statement “LAF helped me to resolve my legal problem to my satisfaction”.

**Fig. 2:**
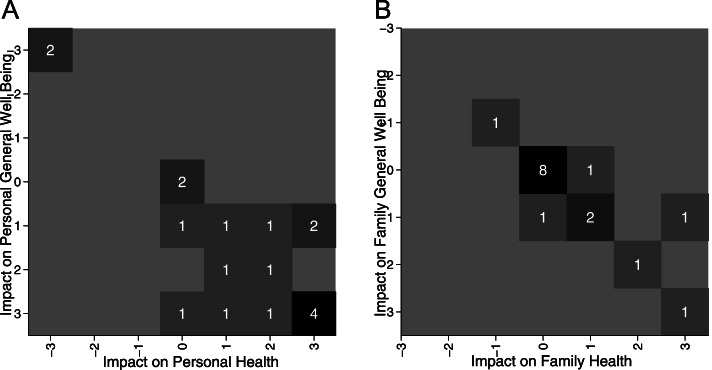
Heat maps of reported impact of legal services on health and general well-being. Legend: Sample of patients in Cook County Health receiving legal services responses (ranging between extremely negative (-3) and extremely positive (+3)) with numbers in parenthesis representing number of respondents for each box. Panel A represents responses for personal health and personal general well-being, and Panel B represents responses for family’s health and family’s general well-being

Eighteen patients responded to questions assessing Legal Aid Chicago services’ impact on respondents’ health or general well-being. These results were visualized on a heat map shown in Fig. 2 Panel (A) The majority reported at least some positive impact on both health and general well-being while 2 respondents reported no impact and 2 respondents reported the most negative impact. Sixteen patients responded to questions assessing Legal Aid Chicago services’ impact on respondents’ family and these results are visualized in the heat map shown in Fig. 2, Panel (B) Report of positive impact on the family was more attenuated than for the patients themselves; however, the majority of those reporting some impact felt the effect was positive.

Eighteen patients provided responses to standardized survey items assessing changes from before to after the legal intervention. In this small-sample analysis, the proportion of patients not reporting “very good” or “excellent” decreased from 16 (89%) prior to the intervention to 8 (44%) following the intervention yielding a risk ratio (95% confidence interval) of 0.20 (0.04, 0.91). Changes in the proportions reporting food insecurity, depression, and high healthcare utilization did not change appreciably as shown in Table 3.

**Table 3:** Relative risk estimate and 95% confidence interval (CI) of reporting adverse health outcomes comparing before and after legal assistance

	Risk Ratio (95% CI)	p-value
Food Insecurity	0.86 (0.63, 1.16)	0.32
General Self Rated Health <=3	0.20 (0.04, 0.91)	0.02
PHQ-2^a^ >= 3	1.00 (0.62, 1.62)	>0.99
2 Hospitalizations over 12 months	0.92 (0.65, 1.31)	0.66
3 ED^b^ visits in 6 months	1.08 (0.72, 1.64)	0.71

^a^Patient Health Questionairre-2

^b^Emergency Department

### Qualitative content analysis

An appraisal of narrative responses further illuminated patients’ experiences. The positive impact of legal services on one’s health was mediated most frequently by provision of instrumental social support, mainly through public benefits like disability entitlements and insurance. Narratives also indicated satisfaction with provision of informational support.

…everyone else was giving me the roundabout and not telling me what is going on until LAF. I was confused at first but they helped me understand every step that is being taken to resolve my issue.

Patients who reported a positive impact on general well-being described, in addition, feelings of being emotionally supported through a stressful process. Iterations of the phrase, “I felt better” recurred in several patient comments.

They helped [me], because [I] was feeling depressed about the situation before.

As to how legal services contributed to patients’ family’s health and well-being, comments mainly pointed to reductions in the family’s anxieties over financial issues.

They don’t have to worry about paying for expensive treatments for me now so they are less stressed.

Individuals who were dissatisfied with our legal services voiced frustrations with accessibility that was somewhat tangential to the quality of services provided. However, customer service quality impacted patient experience in concrete ways as illustrated by the following narrative.

The phone service is hard to get through and they do not return phone calls. I’ve left many messages. When they tell you they will transfer you to people to help you they don’t. They don’t do much to help people with disabilities.

## Discussion

Based on the published literature [6], a typical MLP serving a general population involves screening of patients who present to the clinic for a doctor’s visit. Our medical partnership had care coordinators efficiently screen a large number of patients for health harming legal needs during their contact with patients in complex care program, bond court, and admission to the hospital or emergency department. This method expanded our health care team to spend an extended time with the patient outside of a clinical visit and focus not just on their physical health, but also identify relevant social determinants of health such as health harming legal needs. Just in the first 18 months, our MLP screened tens of thousands of patients in a general high risk population spread across a large urban county. Of patients screened in our population, 1.7% were referred to the MLP, a rate similar to models that were previously described (1-8%) [6]. However, our referral rate in the lower end of the previously established range may represent a novel capacity of trained care coordinators to help resolve some legal issues without involving legal experts. The primary legal concern focused around public benefits (57%), which is similar to the 82% of legal partners in other MLPs who received referrals for income and insurance needs [7].

Our strategy successfully referred to legal services a higher proportion of patients who required the highest level of representation. In our model 30% of cases required “full representation” which Legal Aid Chicago defined as limited action, negotiated settlement (with and without litigation), administrative agency decision, and extensive service. In the Veteran’s Administration only 8.2% of cases required court appearances or hearings [18], and at Lancaster General Hospital in Pennsylvania 16% of disability cases for “super-utilizers” required direct representation [19]. Variations in the percentage of patients needing increased legal support may be based on differences in definitions for full representation. However, as care coordinators are trained to address some issues surrounding benefits such as social security benefits; only more complex cases were referred to the MLP. To complement these skills the medical legal partner trained and provided care coordinators with resources for self advocacy which allowed the care coordinators to make a higher proportion of referrals for legally complex issues. By increasing the proportion of referrals requiring full representation, this allowed the legal partner to focus their resources on more legally complex cases. Another measure of legal aid relates to the duration of services. At the Veterans Administration each legal issue took on average 5.4 h of partnership time [18]. Our partnership measured the length of legal aid in days from referrals to closing the case and we experienced an average numbers of 58 days to closing the case, with a median of 29 days from referral to closure.

Despite the small number of patients who participated in the questionnaire study, there was a statistically significant improvement of General Self Rated Health from the legal interventions. This finding echoes findings of a study in a low-income primary care clinic which reported increased Measure Yourself Concerns and Wellbeing (MYCaW) scores demonstrating improved self-rated health after intervention for health harming legal needs [20]. We further demonstrated an extended impact of the legal intervention on the general well-being of interviewed patients’ family. Financial burden are not shouldered by patients alone and often involve family. The subtle signals of wider benefit may be similar to two previous studies that used Perceived Stress Scale (PSS-10) to evaluate the change in stress before and after legal intervention. The first study demonstrated that reduction in stress in adult patients who had legal intervention by an improvement in the PSS-10 score of 8.1 points [20], and a study in a pediatric clinic demonstrated reduction in stress in parents of children at a pediatric clinic with a reduction in PSS-10 score of 2.5 [21].

The return on investment from the program was substantial and compares favorably to other studies in the literature. Specifically, our 263% return was similar to that seen in a rural medical legal partnership in southern Illinois which demonstrated a ROI of 149% and 221% when measured over two separate study periods [22]. We did not demonstrate a change in utilization of emergency department visits or hospitalizations as a result of the legal intervention.

There are a few limitations to our delivery model. Our model employed team members located outside of the clinic to coordinate the provision of legal resources to patients in order to reduce clinicians’ burden for addressing health harming legal needs. This meant that physicians and clinical staff may not have always been aware of their patients’ health harming legal needs or the targeted interventions. In the absence of training targeting clinicians, there was a lost opportunity to identify and address social determinants of health during routine interaction with a health care system. However, LCSWs located at each clinic did receive training about health harming legal needs and how to refer to the MLP allowing some referrals to come directly from primary care clinics. The impact of divorcing legal assistance from clinical care is unknown, but a more transparent cross-sector information system is necessary to improve communication between team members to ensure comprehensive coordinated care for the patient.

The ongoing rise in the number of referrals indicates that we continue to uncover unmet legal needs that already exceed the capacity in our limited legal staff. The closed loop between Legal Aid Chicago and the referring care coordinator improves the quality of referrals to Legal Aid Chicago to ensure the capacity of the legal staff is focused on more complex legal issues that require full representation. We continue to explore strategies to increase efficiency and protect sustainability through our ongoing efforts. As for limitations to our evaluation, the small number of patients who participated in the survey limited the ability to fully analyze changes to health and utilization of health care services such as use of the emergency department or hospitalizations, though over the short duration of the study we noted a significant return on investment. Our health system’s patient population is fairly unique and our experience may generalize only to a subset of the most vulnerable urban patients in other care settings.

## Conclusions

Training clinicians to recognize health harming legal needs is important component but not a durable strategy for universal screening of patients for health harming legal needs in health care organizations that are already struggling to manage all their competing commitments. In order to develop a sustained plan for screening patients multiple members of the health care team need to be trained and involved in the patient evaluation of social determinants of health and health harming legal needs. In our model, the MLP between Legal Aid Chicago and Cook County Health trained care coordinators to screen for health harming legal needs during their routine screening of patients for general health and social determinants of health and while coordinating care of complex care patients. This technique allowed Cook County Health to provide screening for health harming legal needs in a culturally and geographically diverse population, without adding responsibilities to already resource strained primary and specialty care clinics. During the course of the 18-month study over 29,000 patients were screened and referrals were made for 492 patients with identified civil legal needs. The rates for referral (1.7%), identified health harming legal needs (57% for public benefits), and need for full representation (30%), were similar to values seen in other studies where screening was provided during a clinical visit. In this model, patients also had improvements in self-reported health and improvements in self and family health and general welling being as seen in other studies. Finally, the model provided a positive return on investment (263%) to the health care system, similar to that seen in other medical legal partnerships.

Care coordinators working to screen patients for medical, legal and socials issues that impact patient health provides an opportunity to help alleviate the time and resource constraints that health care clinics face and provide a sustained model to screen large numbers of patients for health harming legal needs. Our rate of referral for “full representation” was higher than previous studies and further studies may be able to determine the ability of care coordinators to address low-complexity legal issues and refer only high-complexity legal issues to a medical-legal partner, and evaluate the role of improved communication between health care team members in assessing and caring for complex patients.

## Data Availability

The datasets generated and/or analyzed during the current study are not publicly available due to potentially patient identifiable information as part of data. However limited data available upon request. Data Requests can be made to Keiki Hinami, MD, Director Applied Research at Cook County Health at khinami@cookcountyhhs.org.

## Abbreviations

MLP: Medical legal partnership
MA: Medical Assistant
LCSW: Licensed Clinical Social Worker
CCH: Cook County Health
I-HELP: Income, Housing & utilities, Education & employment, Legal status, Persinal & family stability
LAF: Legal Aid Foundation (now Legal Aid Chicago)
MYCAW: Measure Yourself Concerns and Wellbeing
ADAPT: services are Advanced Directives, Powers of Attorney for Health and Transfer of Instruments
PSS-10: Perceived Stress Scale
ROI: Return on Investment
